# Gateway Vectors for Simultaneous Detection of Multiple Protein−Protein Interactions in Plant Cells Using Bimolecular Fluorescence Complementation

**DOI:** 10.1371/journal.pone.0160717

**Published:** 2016-08-04

**Authors:** Akane Kamigaki, Kazumasa Nito, Kazumi Hikino, Shino Goto-Yamada, Mikio Nishimura, Tsuyoshi Nakagawa, Shoji Mano

**Affiliations:** 1 Department of Evolutionary Biology and Biodiversity, National Institute for Basic Biology, Okazaki, Japan; 2 Graduate School of Science, Kyoto University, Kyoto, Japan; 3 Department of Cell Biology, National Institute for Basic Biology, Okazaki, Japan; 4 Department of Molecular and Functional Genomics, Interdisciplinary Center for Science Research, Organization for Research, Shimane University, Matsue, Japan; 5 Department of Basic Biology, School of Life Science, SOKENDAI (The Graduate University for Advanced Studies), Okazaki, Japan; CSMCRI, INDIA

## Abstract

Bimolecular fluorescence complementation (BiFC) is widely used to detect protein—protein interactions, because it is technically simple, convenient, and can be adapted for use with conventional fluorescence microscopy. We previously constructed enhanced yellow fluorescent protein (EYFP)-based Gateway cloning technology-compatible vectors. In the current study, we generated new Gateway cloning technology-compatible vectors to detect BiFC-based multiple protein—protein interactions using N- and C-terminal fragments of enhanced cyan fluorescent protein (ECFP), enhanced green fluorescent protein (EGFP), and monomeric red fluorescent protein (mRFP1). Using a combination of N- and C-terminal fragments from ECFP, EGFP and EYFP, we observed a shift in the emission wavelength, enabling the simultaneous detection of multiple protein—protein interactions. Moreover, we developed these vectors as binary vectors for use in Agrobacterium infiltration and for the generate transgenic plants. We verified that the binary vectors functioned well in tobacco cells. The results demonstrate that the BiFC vectors facilitate the design of various constructions and are convenient for the detection of multiple protein—protein interactions simultaneously in plant cells.

## Introduction

BiFC is used to detect protein—protein interactions through visualization in living cells. This technique uses two non-fluorescent fragments derived from a split fluorescent protein, such as GFP or its derivatives. The non-fluorescent fragments are fused to genes encoding proteins or peptides of interest, and the fusion genes are expressed simultaneously in the same cell. Reconstitution of fluorescence takes place only when the two proteins or peptides of interest interact (see reviews; [1–5]). That is, the protein—protein interaction is revealed by the appearance of a reconstituted fluorescence signal. The BiFC technique has become one of the most popular techniques in plant biology [6–15], due to its simplicity, ease of use, and its compatibility with conventional fluorescence microscopy.

When designing vectors expressing proteins fused to a split fluorescent fragment, it is important to consider which ends of the interacting proteins or peptides should be fused to which ends of the N- or C-split fluorescent fragments. There are eight possible combinations (see Fig 1D in [1] and Fig 9C in [5]). When the choice of location of a protein or peptide in a fusion protein is limited to either terminus of a split fluorescent protein, fewer combinations need to be tested, since improper fusion to a split fragment alters protein function and/or eliminates information about subcellular targeting. For example, the peroxisome targeting signal 2 (PTS2) works only when it is located at the N-terminal end of a protein [12]. That is, since PTS2 must be located in front of the split fragment, it is not necessary to generate fusion genes in which PTS2 is located at the C-terminus of a split fragment; thus, which results in four possible combinations. However, if no information is available about protein function or subcellular localization, sometimes all eight combinations must be tested. In such cases, fusion gene construction is time-consuming. To date, several BiFC vectors have been developed for use in living cells [6, 7, 14, 16–20]. These vectors can be divided into two groups based on the method used to insert the DNA fragment. One type of vector contains a multicloning site for use with restriction enzymes and ligase. The other type utilizes a recombination reaction based on Gateway cloning technology [7, 14]. Gateway cloning technology, available through Thermo Fisher Scientific, has become one of the most popular methods for manipulating DNA fragments, because various fusion genes can be constructed easily and simultaneously. We previously developed a Gateway cloning technology-compatible vector construction system and continue to provide various destination vectors that are useful in plant research [5, 14, 21–23]. Here, we demonstrate the utility of additional, newly developed Gateway cloning technology-compatible BiFC vectors. These BiFC vector sets have the following advantages: they are (1) suitable for the rapid, simple construction of many fusion genes; (2) easily accommodate constructions with four different types of fluorescent proteins to detect simultaneously multiple protein—protein interactions using protein fusions to various combinations of split fragments; and (3) enable the use of transient expression systems such as particle bombardment assays, the Agrobacterium infiltration method, and transgenic plant generation, since two types of vector sets are prepared, including vectors for transient expression and binary vectors.

## Materials and Methods

### Plasmid construction to generate Gateway-compatible BiFC vectors

New BiFC vectors employing ECFP and EGFP for transient expression were constructed using the strategy previously employed for EYFP-based BiFC vector construction [14] using the same primers to amplify *ECFP* and *EGFP* cDNA fragments and the same vector backbones, pUGW0 and pUGW2, resulting in the generation of pGWnC, pGWnG, pGWcCG, pnCGW, pcCGW and pcCGGW. Since the nucleotide sequences of the C-terminal fragments of CFP and GFP are the same, this sequence was designated cCG.

*mRFP1* cDNA [24] was used to construct RFP-based vectors. Nucleotide substitutions were introduced at nucleotides 196 and 197, leading to a Gln to Thr substitution, because this substitution enhances the intensity of fluorescence [25]. The DNA fragment corresponding to the N-terminal region (Met^1^–Asp^154^) of mutated mRFP1 (nRFP) was amplified with primers mRFP1BN-F and mRFP1BN-Rter and introduced into the *Aor*51HI site of pUGW2 [21], yielding pGWnR. The nRFP fragment was amplified with primers mRFP1BN-F and mRFP1BN-R, and introduced into the *Aor*51HI site of pUGW0 [21], yielding pnRGW. The C-terminal region (Gly^155^–Lys^225^) of mutated mRFP1 (cRFP) was amplified with primers mRFP1BC-F and mRFP1BC-RTer and introduced into the *Aor*51HI site of pUGW2, yielding pGWcR. The cRFP fragment amplified with primers mRFP1BC-Fmet and mRFP1BC-R was introduced into the *Aor*51HI site of pUGW0, yielding pcRGW. The nucleotide sequences encoding ten amino acid residues of the myc-epitope (Glu-Gln-Lys-Leu-Ile-Ser-Glu-Glu-Asp-Leu) and HA-epitope (Lys-Pro-Lys-Asp-Val-Pro-Asp-Tyr-Ala-Gly) were conjugated at the 5’ end of N-terminal and C-terminal RFP fragments, respectively. The nucleotide sequences of primers used to amplify *mRFP1* cDNA fragments are shown in S1 Table. *E*. *coli* DB3.1 (Thermo Fisher Scientific, Yokohama, Japan) cultures harbouring each vector were selected on LB medium containing 50 mg/l ampicillin and 30 mg/l chloramphenicol.

To construct the binary vectors, the Gateway cassette and split fluorescent protein genes, which were digested and purified from pGWnX, pGWcX, pnXGW and pcXGW (X indicates C, G, Y and R of CFP, GFP, YFP and mRFP1, respectively), were introduced into pGWB400 or pGWB500 as described previously [21] to produce pB4GWnX, pB4GWcX, pB4nXGW, pB4cXGW, pB5GWnX, pB5GWcX, p54nXGW, and pB5cXGW. *E*. *coli* DB3.1 (Thermo Fisher Scientific, Yokohama, Japan) cultures harbouring these vectors were selected on LB medium containing 50 mg/l spectinomycin and 30 mg/l chloramphenicol. The PCR-amplified regions and ligation junctions were confirmed by sequence analysis of all vectors. The BiFC vectors are available for the expression of proteins (designated Z) fused with a split XFP (nXFP and cXFP, the N- or C-terminal fragment, respectively, of CFP, GFP, YFP and RFP) fragment under the control of the cauliflower mosaic virus 35S promoter, including pGWnY for Z-nYFP, pnYGW for nYFP-Z, pGWcY for Z-cYFP and pcYGW for cYFP-Z.

### Construction of fusion genes

To construct *nXFP-PEX7*, *PTS2-cXFP* (nXFP and cXFP, corresponding to the N- and C-terminal fragments, respectively, of CFP, GFP, YFP and RFP) and *nRFP-PEX12* fusion genes, corresponding to the entry clones containing cDNAs encoding full-length PEX7 [26], PEX12 [27], or PTS2-containing 48 N-terminal amino acids of pumpkin citrate synthase [28], were subjected to LR recombination using the appropriate destination vectors. The combinations of entry clones and destination vectors are shown in S2 Table.

### Particle bombardment

Two types of plasmids in various combinations were introduced simultaneously into onion epidermal cells using a Helios Gene Gun (BIO-RAD, Tokyo, Japan) as described previously [27]. Four types of constructs were used to detect simultaneously multiple protein−protein interactions, and four types of plasmids, each containing one-fourth of the total DNA, were mixed prior to use.

### Transient gene expression by Agrobacterium infiltration

Wild tobacco plants (*Nicotiana benthamiana*) were grown at 28°C. Agrobacterium cultures harbouring the fusion gene of interest was grown overnight in LB media containing 50 mg/l spectinomycin at 28°C and centrifuged at 4,000 x *g* for 15 min. Cell pellets were resuspended in infiltration buffer (10 mM MES, 10 mM MgCl_2_). Appropriate pairs of cell suspensions were mixed, and their concentrations were adjusted to OD_600_ = 0.5 to 0.6. The cell suspensions were infiltrated into 6-week-old *Nicotiana benthamiana* leaves with syringes, followed by incubation for 3 days. The leaves were observed by confocal microscopy.

### Microscopic observation

Plant tissues were examined under an LSM510 META laser scanning confocal microscope equipped with Argon and HeNe lasers (Carl Zeiss, Tokyo, Japan). To detect the signals from CFP, GFP, YFP, and mRFP1, emission filters BP480-520, BP500-530, BP535-590, and BP560-615 were used, respectively. The lambda acquisition mode of the LSM510 META system was used to determine the spectra.

### Immunoblot analysis

Agrobacterium-infiltrated wild tobacco leaves were homogenized in extraction buffer (20 mM Tris-HCl, pH 6.8, 50 mM DTT, 2% SDS, and 24% glycerol) and boiled for 5 min. The homogenates were centrifuged at 20,400 x *g* for 15 min at 4°C and subjected to SDS-PAGE in 12.5% polyacrylamide gels, followed by transfer to PVDF membranes (Merck Millipore, Darmstadt, Germany) using a semidry electroblotting system. Myc-tagged nRFP and HA-tagged cRFP were detected using Myc (MBL, Nagoya, Japan) and HA antibodies (Convance Japan, Tokyo, Japan), respectively. The immunologic reactions were monitored by detecting the activity of horseradish peroxidase-coupled antibodies against mouse IgG (ECL system; GE Healthcare Japan, Tokyo, Japan).

### Nucleotide accession numbers

The nucleotide sequences reported in this paper have been registered in GenBank/EMBL/DDBJ under accession numbers AB830536–AB830573.

## Results

### Construction of pUGW-based Gateway cloning technology-compatible vectors for BiFC

**Fig 1:**
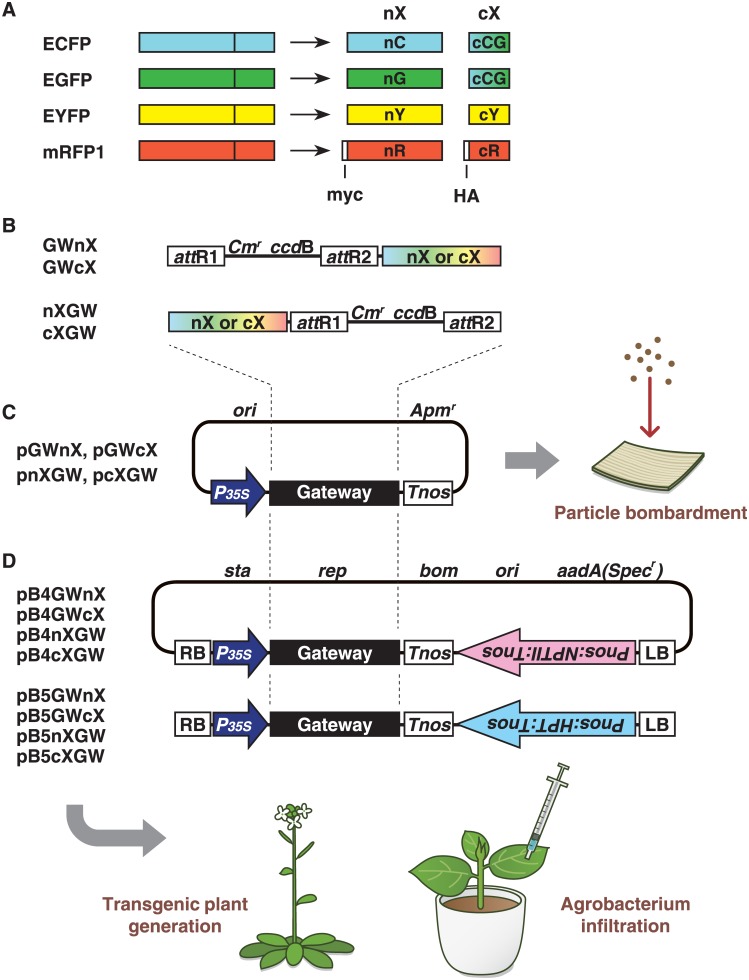
Schematic diagrams of the Gateway cloning technology-compatible vectors. A. ECFP, EGFP, EYFP and mRFP1 can be divided into two fragments. The letters ‘n’ and ‘c’ represent N- or C-terminal fragments of a split fluorescent protein, and the letters ‘C’, ‘G’, ‘Y’ and ‘R’ represent the type of fluorescent protein (ECFP, EGFP, EYFP or mRFP1). Since the nucleotide sequences of C-terminal CFP and C-terminal GFP are identical, we designated the fragment cCG. The letters ‘myc’ and ‘HA’ in the N- and C-terminal fragment from mRFP1 represent myc- and hemagglutinin-epitope tags, respectively. B. The structures of the region indicated as ‘Gateway’ in (C) and (D). GWnX and GWcX contain N- or C-terminal split fluorescent protein downstream of the *att*R2 site, respectively, whereas nXGW and cXGW contain N- or C-terminal split fluorescent protein upstream of the *att*R1 site, respectively. C. Outline of the pUGW-based vectors for BiFC. pGWnX, pGWcX, pnXGW, and pcXGW vectors, which bear the DNA fragment shown in (B) downstream of the 35S promoter from cauliflower mosaic virus. D. Outline of the binary vectors for BiFC. The pB4 and pB5 series contain Km^r^ and Hyg^r^ markers, respectively, which are placed in reverse orientation to the genes cloned via LR recombination. Details of plasmid construction and the vector backbone are given in Materials and Methods. *Cm*^*r*^, chloramphenicol-resistance marker; *ccd*B, negative selection marker used in bacteria; *35Sp*, 35S promoter; *Tnos*, nopaline synthase terminator; myc, c-myc affinity tag; HA, hemagglutinin affinity tag.

We applied our pUGW-based Gateway cloning technology-compatible vector construction system, which uses vectors derived from pUC119 [14, 21, 22], to generate destination vectors for BiFC containing each split fluorescent fragment. We previously reported the construction of four EYFP-based BiFC vectors, pGWnY, pGWcY, pnYGW and pcYGW, using this system [14]. The same method was adopted to construct ECFP-, EGFP- and mRFP1-based BiFC vectors (Fig 1A). The N- and C-terminal fragments of ECFP and EGFP were amplified using the same primers, which were used for amplification of N- and C-terminal fragments of EYFP, since the nucleotide sequences at the positions used to split a fluorescent protein into two fragments in ECFP, EGFP and EYFP are identical. To amplify the mRFP1 fragments, we prepared seven primers based on the nucleotide sequence of mRFP1 [24] (S1 Table). The PCR products were subcloned into the *Aor*51HI sites of the parental vectors, pUGW2 or pUGW0, respectively, generating vectors pGWnX, pGWcX, pnXGW, and pcXGW (Fig 1B). The letters ‘n’ and ‘c’ in the name of each vector represent N- or C-terminal fragments of a split fluorescent protein, and ‘X’ represents the type of fluorescent protein (ECFP, EGFP, EYFP or mRFP1). The order in which nX/cX and GW are listed shows the order of a split fluorescent fragment and the Gateway cassette. For example, the Gateway cassette is located in front of the N-terminal ECFP fragment in the pGWnC vector. This rule applies to all of our BiFC vectors. Since the nucleotide sequences of the C-terminal CFP and GFP are exactly the same, we designated the vectors pGWcCG or pcCGGW. In addition to the construction of the four EYFP-based BiFC vectors, we produced 10 new BiFC vectors.

One problem with BiFC is that the reconstituted signals are not always observed. There are two possible reasons for this. In the first case, reconstitution may not occur, although two fusion genes are correctly expressed in the same cell. In this case, we determine that the two proteins of interest do not interact. In the second case, one or both fusion genes are not expressed correctly. To distinguish between these two possibilities, immunoblotting can be used to detect the production and accumulation levels of fusion proteins. Therefore, we added myc- and hemagglutinin (HA)-epitope tags to the 5’-ends of N- and C-terminal mRFP1 fragments, respectively (Fig 1A), because it is easy to obtain good commercial antibodies for these epitopes. Indeed, we detected fusion proteins using antibodies against these epitopes, as described below.

### Construction of binary vectors and application to *Agrobacterium* infiltration

**Fig 2:**
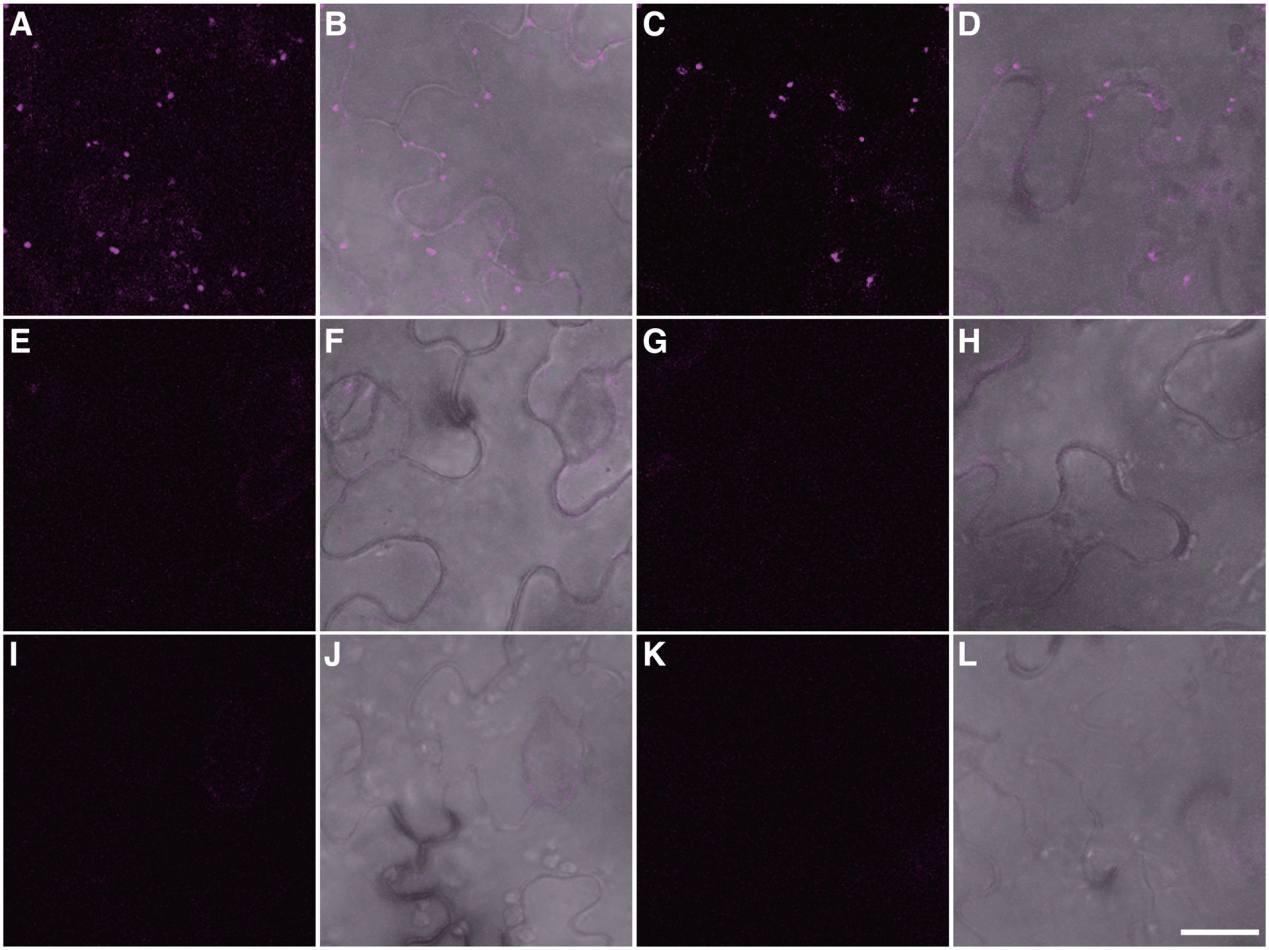
Detection of protein—protein interactions between PMP38–PMP38 and PEX7–PTS2 in tobacco leaves by Agrobacterium infiltration. Various combinations of fusion genes were introduced by Agrobacterium infiltration. (A, C, E, G, I and K) Detection of red fluorescent signals from reconstituted mRFP1. (B, D, F, H, J and I) Merged images of reconstituted fluorescent signals with differential interference contrast settings. (A, B) PMP38-nRFP with PMP38-cRFP. (C, D) nRFP-PEX7 with PTS2-cRFP. (E, F) nRFP-PEX7 with PMP38-cRFP. (G, H) PMP38-nRFP with cRFP-PEX12. (I, J) PMP38-nRFP with untagged cRFP. (K, L) nRFP-PEX7 with untagged cRFP. Bar: 20 μm.

We developed the BiFC vectors as binary vectors to make them suitable for Agrobacterium infiltration and for generating transgenic plants using our conversion system to produce binary vectors [21]. We generated 28 binary vectors for BiFC, half containing a kanamycin resistance gene (pB4 series) and the other half containing a hygromycin resistance gene (pB5 series) as selection markers in plants (Fig 1D). To verify the utility of these binary vectors for *in vivo* BiFC assays, we carried out Agrobacterium infiltration to detect the homooligomerization of peroxisomal membrane protein 38 (PMP38)–PMP38, and the interaction of PEX7, the receptor for PTS2-containing proteins, with PTS2. The interaction between these proteins was previously identified by yeast two-hybrid analysis, and EYFP-based BiFC was previously performed on these proteins, which were transiently expressed in onion epidermal cells [12, 15]. We mixed two *Agrobacterium tumefaciens* cultures harbouring *PMP38-nRFP* with *PMP38-cRFP*, or *nRFP-PEX7* with *PTS2-cRFP*, and then co-infiltrated these cultures into the leaf cells of *Nicotiana benthamiana*. Reconstituted RFP signals were observed as punctate structures in the case of PMP38–PMP38 (Fig 2A and 2B) and PEX7–PTS2 (Fig 2C and 2D) interactions. When different combinations were used, such as PMP38 with PEX7 and PMP38 with PEX12, one of peroxisomal membrane proteins [27], reconstituted signals were not observed, although minor, faint background signals were detected (Fig 2E, 2F, 2G and 2H). For the other negative controls, we co-infiltrated *PMP38-nRFP* or *nRFP-PEX7* with untagged *cRFP*. As expected, reconstituted fluorescence was not observed (Fig 2I, 2J, 2K and 2L). These results demonstrate that our binary vectors functioned well when used in Agrobacterium infiltration.

**Fig 3:**
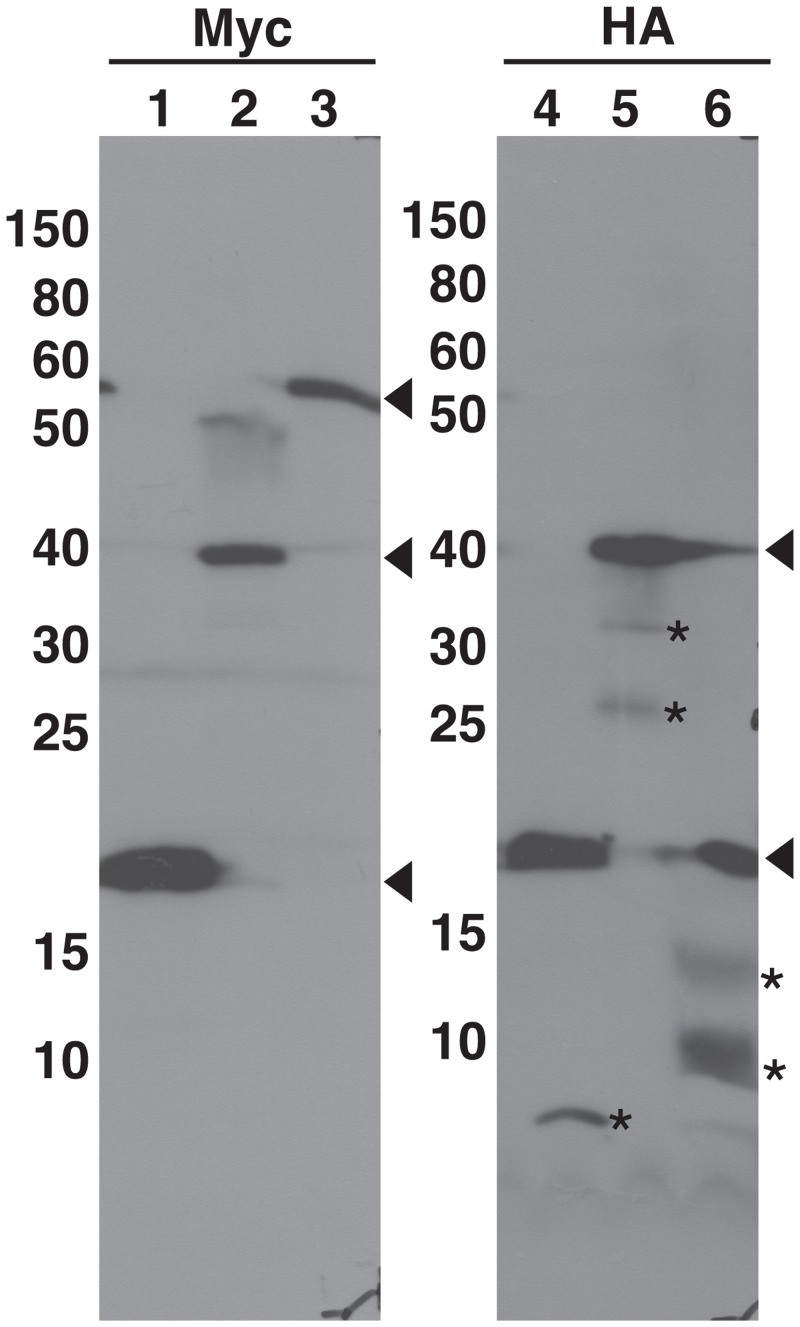
Immunodetection of transiently expressed nRFP/cRFP-fused proteins. Using protein extracts prepared from tobacco leaves of Agrobacterium-infiltrated plants analyzed in Fig 2, the accumulation of nRFP/cRFP-fused proteins by Agrobacterium infiltration was confirmed by immunoblot analysis with anti-Myc or anti-HA antibodies. Lane 1, nRFP; Lanes 2 and 5, PMP38-nRFP with PMP38-cRFP; Lanes 3 and 6, nRFP-PEX7 with PTS2-cRFP; Lane 4, cRFP. Arrowheads indicate the positions of untagged or tagged polypeptides. Asterisks represent extra polypeptides, which are considered to be degradation products of the fusion proteins.

As stated above, it is sometimes necessary to determine the expression levels of fusion proteins. The mRFP1-based BiFC vectors contain myc- and HA-epitope tags at the 5’-ends of N- and C-terminal fragments, respectively (Fig 1B). Using protein extracts prepared from leaves of Agrobacterium-infiltrated plants analyzed in Fig 2, immunoblot analysis was carried out to verify whether these epitope tags function well for the detection of fusion proteins (Fig 3). We detected immune-reactive bands that correspond to the expected sizes of the fusion proteins (Lanes 2, 3, 5 and 6 in Fig 3), although some extra bands, which are considered to be due to degradation of fusion proteins, were also detected. Myc-nRFP and HA-cRFP (split RFP proteins without fusions) were also detected (Lanes 1 and 4 in Fig 3). These results show that the Myc- and HA-epitope tags functioned correctly in this assay.

### Evaluation of fluorescence properties among different combinations of fragments

**Fig 4:**
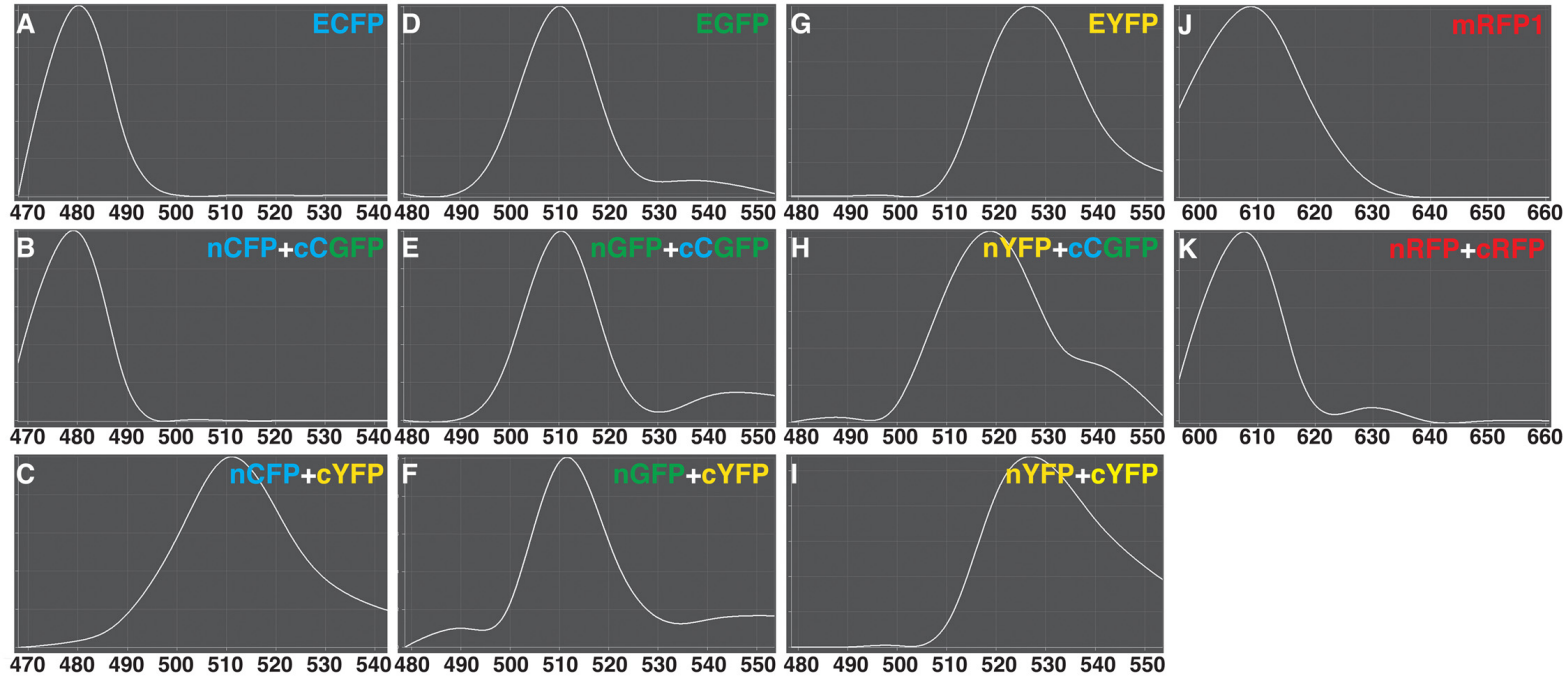
Fluorescence emission spectra of intact and reconstituted fluorescent proteins. Using the interaction between PEX7 and PTS2 as one of the split combinations transiently expressed in onion epidermal cells, florescence emission spectra were measured following excitation with an argon laser at 458 nm (A–L) or a HeNe laser at 543 nm (J, K) using the LSM510 META system. X- and Y-axes represent wavelength and relative fluorescence intensity, respectively. The maximum emission of each spectrum was taken as 1.0, and in each curve, fluorescence at a given wavelength was normalized relative to the maximum. (A) non-split ECFP, (B) nCFP with cCGFP, (C) nCFP with cYFP, (D) non-split EGFP, (E) nGFP with cCGFP, (F) nGFP with cYFP, (G) non-split EYFP, (H) nYFP with cCGFP, (I) nYFP with cYFP, (J) non-split mRFP1 and (K) nRFP with cRFP.

To verify the utility of our BiFC vectors, we examined the spectral properties of fluorescence emissions using the interaction between PEX7 and PTS2 among the various split combinations. We prepared various combinations of nXFP-PEX7 with PTS2-cXFP (nXFP and cXFP, corresponding to the N- or C-terminal fragment, respectively, of CFP, GFP and YFP), and carried out particle bombardment experiments using onion epidermal cells. S1 Fig shows a representative result using nYFP-PEX7 with PTS2-cCGFP. The punctate fluorescent pattern, indicating peroxisomes, was in good agreement with previous results [5, 12], since PEX7-PTS2 complexes are localized to peroxisomes after interaction in the cytosol. Then, we measured the spectral properties of fluorescence emissions from intact and reconstituted fluorescent proteins to substantiate the reconstitution of the chromophore of each fluorescent protein by examining the interaction among various combinations using the lambda acquisition mode of the LSM510 META system (Fig 4). The patterns of fluorescence emission spectra (between 470 and 540 nm) of intact and reconstituted ECFPs were almost identical, revealing an emission maximum at 480 nm (Fig 4A and 4B). However, when the C-terminal fragment of EYFP was used in place of the C-terminal fragment ECFP, the pattern of the emission spectrum changed; the emission maximum occurred at 510 nm (Fig 4C). For EGFP, we investigated the pattern of fluorescence emission spectra between 480 and 550 nm, revealing that the emission spectrum pattern and emission maximum at 510 nm in intact versus reconstituted EGFPs were almost identical (Fig 4D and 4E). Curiously, using the C-terminal fragment of EYFP in place of the C-terminal fragment of EGFP did not cause the large shift in emission spectra, which occurred using other combinations (Fig 4F). For EYFP, the patterns of fluorescence emission spectra between 480 and 550 nm were almost identical between non-split and reconstituted EYFPs, revealing an emission maximum at 525 nm (Fig 4G and 4I). Using the C-terminal fragment of ECFP in place of the C-terminal fragment EYFP caused the pattern of emission spectra to shift, with an emission maximum observed at 519 nm (Fig 4H). For mRFP1, we investigated the pattern of fluorescence emission spectra between 595 and 660 nm in intact versus reconstituted mRFP1. An emission maximum was observed at 609 nm for both types of protein. The emission spectrum patterns were almost identical, although reconstituted red fluorescence had a slightly higher emission spectra at 630 nm than that of non-split mRFP1 (Fig 4J and 4K). These results demonstrate that the BiFC vectors can potentially be used for multicolor BiFC to detect multiple protein—protein interactions simultaneously, if different combinations of fragments are used (see later).

### Simultaneous detection of two protein–protein interactions

**Fig 5:**
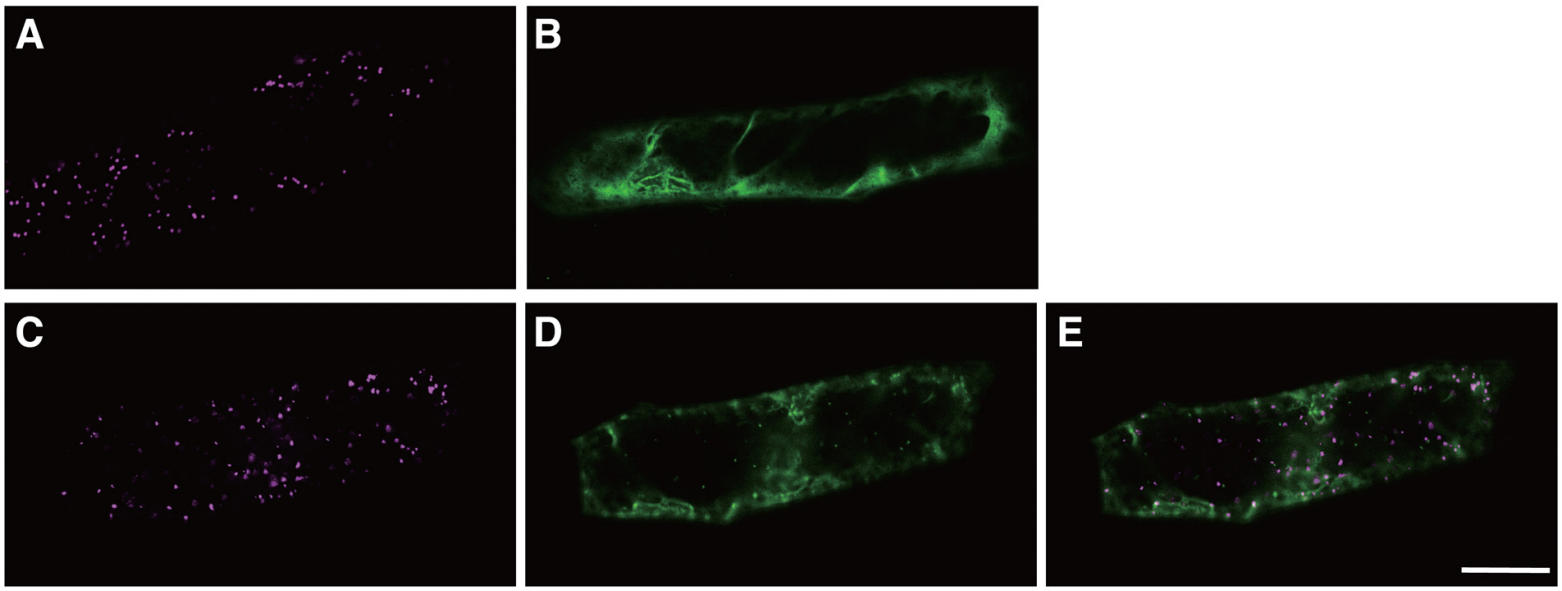
Detection of multiple protein—protein interactions using transient expression assays. (A, B) Two types of vectors were introduced as controls. Representative images of interactions of nRFP-PEX7 with PTS2-cRFP (A) and AtSEC31A-nYFP with AtSEC13A-cYFP (B). (C-E) Four types of vectors, nRFP-PEX7, PTS2-cRFP, AtSEC31A-nYFP, and AtSEC13A-cYFP, were introduced simultaneously into onion epidermal cells. Representative images of interactions of nRFP-PEX7 with PTS2-cRFP (C) and AtSEC31A-nYFP with AtSEC13A-cYFP (D) in the same cell. (E) Merged image of (C) with (D). Bar: 50 μm.

To obtain insights into multiple protein—protein interactions by examining different fluorescent proteins in a single cell, we tried to detect two interactions by simultaneously introducing four types of constructs, *nRFP-PEX7*, *PTS2-cRFP*, *AtSEC31A-nYFP* and *AtSEC13A-cYFP*, into onion epidermal cells by particle bombardment. AtSec31A and AtSec13A are components of COP II vesicles, which mediate protein transport from the endoplasmic reticulum to the Golgi apparatus, and the interaction between these two proteins has been previously reported using the BiFC assay [14]. Signals from reconstituted mRFP1 and EYFP were detected in the same cells (Fig 5C, 5D and 5E). The punctate signals from reconstituted mRFP1 indicate the presence of peroxisome-localized PEX7–PTS2 interactions (Fig 5C), and the cytosolic signals from reconstituted EYFP indicate the presence of AtSEC31A—AtSEC13A interactions (Fig 5D). These mRFP1- and EYFP-based signals obviously exhibited different localizations (Fig 5E) in the same cells. The detection of these signals from reconstituted mRFP1 or EYFP is in good agreement with the results obtained using only two construct combinations, i.e., nRFP-PEX7 with PTS2-cRFP (Fig 5A) and AtSEC31A-nYFP with AtSEC13A-cYFP (Fig 5B), as controls. These results indicate that simultaneous protein—protein interactions can be visualised as different colours of fluorescence in a single cell.

## Discussion

In this study, we demonstrated that it was possible to make an efficient construction system for the generation of various fusion genes for the BiFC assay. To date, several vectors have been developed for use in the BiFC assay in living cells [6, 7, 14, 17–20, 29]. The types of fluorescent proteins utilized with our vector series are not novel, since ECFP, EGFP, EYFP and mRFP1 have previously been used in BiFC assays. However, our BiFC vectors are quite useful, because they are compatible with Gateway cloning technology, leading to easier and more convenient construction of various fusion genes at the same time, unlike the conventional method, which requires the use of restriction enzymes, ligases, and subcloning. In addition, our BiFC vectors contain *att*R1 and *att*R2 sites, which are required for the LR recombination reaction (Fig 1B). Since most users of Gateway cloning technology generate entry clones containing the *att*L1 and *att*L2 sites, such entry clones can be used to construct fusion genes using our BiFC vectors without any modifications.

Most BiFC vectors used for Agrobacterium infiltration to date require digestion with restriction enzymes and ligation. Although Gateway cloning technology has been used to clone genes into some previously described vectors [7], these vectors were not designed to be used with a variety of fluorescent proteins. The new binary vector series enables the use of various combinations of four types of fluorescent proteins, extending the range of choices for users. Our BiFC vector series will be useful for carrying out BiFC assays in transgenic plants expressing fluorescent proteins, because users can use vectors encoding proteins producing fluorescent signals of different colors. In addition, our binary vectors contain either kanamycin or hygromycin resistance genes. Therefore, users will be able to conduct protein-protein interaction assays using stable transgenic plants expressing both N- and C-terminal fragments, because such transgenic plants can be easily obtained by screening on medium containing both antibiotics when transgenic plants expressing either the N- or C-terminal fragment are crossed. The BiFC vectors in this study have been already distributed to other laboratories, where they are being used in plant research [30, 31]. Currently, we are using these vectors to investigate the interactions between novel peroxisomal proteins.

### Detection of protein–protein interactions simultaneously using the multicolor BiFC assay

To date, the multicolor BiFC assay has been used with various fluorescent proteins [16, 18, 20, 29, 32]. There are two types of the multicolor BiFC assays. One type is used to detect interactions between one common component and two interactors, such as the CIPK24–CBL1 and CIPK24–CBL10 interactions using Venus and CFP in the same cell by introducing three types of vectors [18]. Users will be able to employ a similar strategy using our BiFC vector combinations, i.e., nCFP/cCGFP with nGFP/cCGFP, nCFP/cCGFP with nCFP/cYFP, or nGFP/cYFP with nYFP/cYFP, because their florescence spectra can be distinguished using a confocal scanning microscope equipped with a spectrum separation device, such as the LSM510 META system (Fig 4).

The other type of multicolor BiFC assay is used to detect proteins that do not share a common component. In this case, users should select fluorescent proteins with large differences in emission spectra, such as GFP and RFP. By introducing four types of vectors for CFP, GFP or YFP with RFP detection, users will be able to detect fluorescent signals from CFP, GFP or YFP with RFP in the same cell without the need for a special spectrum separation device. Indeed, we succeeded in detecting two interactions, i.e., PEX7–PTS2 in peroxisomes and AtSEC31A—AtSEC13A in the cytosol, by detecting YFP and mRFP1 signals (Fig 5).

### Points to consider when using our BiFC vectors and future perspectives

Although BiFC is a simple and convenient technique, artificial noise is a potential problem. To alleviate this problem, it is essential to use a negative control vector, such as an untagged vector (Fig 2I, 2J, 2K and 2L) and/or a vector containing a gene proven not to interact with the protein (Fig 2E, 2F, 2G and 2H), in the BiFC assay. However, this measure may not be sufficient. As shown in S2 Fig, in some cases, signals are produced indicating even when using non-interacting combinations of proteins. Therefore, the detection of interactions by the BiFC assay should be verified using other approaches such as immunobloting, if possible. As shown in Fig 3, the Myc-tag and HA-tag in our mRFP1-based BiFC vectors worked correctly to detect fusion proteins. Similar results have been reported using other antibodies against Glu-Glu tag and GFP in addition to Myc- and HA-tags [6, 7, 18]. However, care should be exercised in interpreting the results from these analyses. Because this assay uses extracts from many cells, and not from one cell, in the infiltrated region, we could not exclude the possibility that each fusion gene expresses in different cells in the infiltrated region.

Future work will include expressing genes in the BiFC vectors under the control of specific promoters to examine the tissue or developmental stage specificity of protein—protein interactions, and constructing a system allowing the expression of both N- and C-terminal-fused genes in one vector, which should facilitate the introduction of DNA into cells. In addition, since a variety of fluorescent proteins with more useful properties, such as strong fluorescence intensity and more rapid formation of chromophores, have become available, a variety of new, excellent BiFC vectors will be generated by incorporating these new fluorescent proteins into the Gateway cloning technology system. Moreover, several studies including those employing structure analysis of fluorescent proteins have shown that the signal-to-noise ratio differs depending on the location of the split site in the fluorescent protein, and that amino acid substitutions in fluorescent proteins can enhance the intensity of the BiFC signal [25, 33–36]. Although we employed split positions located between the 154th and 155th amino acid in mRFP1, it was reported that the split between these amino acids generated no detectable reconstituted fluorescence [25]. This inconsistency might be due to the insertion of amino acids, which are derived from *att* sequences, between a split fluorescent fragment and a fused protein. Other split positions have been reported to result in stronger fluorescence [25]. In the future, we will investigate the effects of other split sites and other amino acid substitutions to improve the BiFC vectors, with the aim of improving fluorescent intensity and the signal-to-noise ratio.

## Supporting Information

S1 Fig Detection of interaction between PEX7 and PTS2 using transient expression assays. Onion epidermal cells were used for particle bombardment experiments using various combinations of nXFP-PEX7 with PTS2-cXFP (nXFP and cXFP, the N- or C-terminal fragment, respectively, of CFP, GFP and YFP). (A-C) Images using the combination of nYFP-PEX7 with PTS2-cCFP are shown as representative data. (A) Reconstituted fluorescence image. (B) Differential interference contrast image. (C) Merged image of (A) with (B). Bar: 50 μm.
(EPS)

S2 Fig Detection of protein—protein interactions by Agrobacterium infiltration. Various combinations of fusion genes were transiently introduced by Agrobacterium infiltration. (A, C, E, and G) Detection of red fluorescent signals from reconstituted mRFP1. (B, D, F and H) Merged images of reconstituted fluorescent signals with differential interference contrast. (A, B) nRFP-PEX7 with cRFP-PEX12. (C, D) PMP38-nRFP with PTS2-cRFP. (E, F) Untagged nRFP with untagged cRFP. (G, H) Untagged nRFP with PMP38-cRFP. Bar: 20 μm.
(EPS)

S1 Table Primer sequences used for PCR to amplify *mRFP1* fragments. (DOCX)

S2 Table Entry clones and destination vectors for LR recombination reaction to generate fusion genes in this study. (DOCX)
