# Supplementary material for: Gateway Vectors for Simultaneous Detection of Multiple Protein−Protein Interactions in Plant Cells Using Bimolecular Fluorescence Complementation
Source: PLoS One. 2016 Aug 4;11(8):e0160717. doi: 10.1371/journal.pone.0160717 (PMC4973907; doi:10.1371/journal.pone.0160717)
Supplement: S1 Table — (DOCX) [file pone.0160717.s003.docx]

| **S1 Table. Primer sequences used for PCR to amplify *mRFP1* fragments** | |
| --- | --- |
| **Primer name** | **Nucleotide sequence** |
| mRFP1BN-F | 5'-ATGGAACAAAAGCTAATCTCCGAGGAAGACTTGATGGCCTCCTCCGAGGACG-3' |
| mRFP1BN-Rter | 5'-TTAGTCCTCGGGGTACATCCG-3' |
| mRFP1BN-R | 5'-GTCCTCGGGGTACATCCG-3' |
| mRFP1BC-F | 5'-TACCCATACGATGTTCCTGACTATGCGGGCGCCCTGAAGGGCGAG-3' |
| mRFP1BC-Fmet | 5'-ATGTACCCATACGATGTTCCTGACTATGCGGGCGCCCTGAAGGGCGAG-3' |
| mRFP1BC-Rter | 5'-TTAGGCGCCGGTGGAGTGGCG-3' |
| mRFP1BC-R | 5'-GGCGCCGGTGGAGTGGCG-3' |
| Underline and double underlines represent nucleotide sequences for myc and hemagglutinin, respectively | |
