# Supplementary material for: Gateway Vectors for Simultaneous Detection of Multiple Protein−Protein Interactions in Plant Cells Using Bimolecular Fluorescence Complementation
Source: PLoS One. 2016 Aug 4;11(8):e0160717. doi: 10.1371/journal.pone.0160717 (PMC4973907; doi:10.1371/journal.pone.0160717)
Supplement: S2 Table — (DOCX) [file pone.0160717.s004.docx]

| **S2 Table. Entry clones and destination vectors for LR recombination reaction to generate fusion genes in this study** | | | | |
| --- | --- | --- | --- | --- |
| **Entry clone** | **Destination vector** | **Fusion gene** | **Plasmid name after LR recombination reaction** | **Use in this study** |
| pDONRPEX7^*^ | pnCGW | nCFP-PEX7 | pnCPEX7 | Particle bombardment |
| pDONRPEX7^*^ | pnGGW | nGFP-PEX7 | pnGPEX7 | Particle bombardment |
| pDONRPEX7^*^ | pnYGW | nYFP-PEX7 | pnYPEX7 | Particle bombardment |
| pDONRPEX7^*^ | pnRGW | nRFP-PEX7 | pnRPEX7 | Particle bombardment |
| pPTS2-221^*^ | pGWcCG | PTS2-cCFP | pPTS2cCG | Particle bombardment |
| pPTS2-221^*^ | pGWcY | PTS2-cYFP | pPTS2cY | Particle bombardment |
| pPTS2-221^*^ | pGWcR | PTS2-cRFP | pPTS2cR | Particle bombardment |
| pDONRPMP38^**^ | pB5GWnR | PMP38-nRFP | pB5PMP38nR | Agrobacterium infiltration |
| pDONRPMP38^**^ | pB5GWcR | PMP38-cRFP | pB5PMP38cR | Agrobacterium infiltration |
| pDONRPEX7^*^ | pB5nRGW | nRFP-PEX7 | pBnRPEX7 | Agrobacterium infiltration |
| pPTS2-221^*^ | pB5GWcR | PTS2-cRFP | pB5PTS2cR | Agrobacterium infiltration |
| p221APM4ter^***^ | pB5cRGW | cRFP-PEX12 | pB5cRPEX12 | Agrobacterium infiltration |
| ^*^These entry clones and plasmids were generated in previous experiments (Tanuja *et al.*, 2009).  ^**^This entry clone was described previously (Mano *et al.*, 2011).  ^***^This entry clone contains the full-length PEX12 cDNA (Mano *et al.*, 2006). | | | | |
